# Effects on Sperms’ Quality of Selegiline in Aged Rats

**DOI:** 10.2174/1874104501711010138

**Published:** 2017-11-30

**Authors:** Huba Kalász, Julianna Thuróczy, Gellért Karvaly, Lajos Balogh, István Gyertyán, Edit Tóth-Molnár, Ernest Adeghate, Kornélia Tekes

**Affiliations:** 1 Department of Pharmacology and Pharmacotherapy, Semmelweis University, 1089 Budapest, Nagyvárad tér 4, Hungary; 2 Animal Health Center Budafok, H-1221 Budapest, Kossuth Lajos Str. 52. Hungary; 3 Department of Laboratory Medicine, Semmelweis University, 1089 Budapest, Nagyvárad tér 4, Hungary; 4 National “FJC” Research Institute for Radiobiology and Radiohigiene, H-1221 Budapest, Anna Str. 5. Hungary; 5 Department of Ophthalmology, University of Szeged, 6720 Szeged, Korányi fasor 10- 11, Hungary; 6 Department of Anatomy, United Arab Emirates University, P.O.Box 17666, Al Ain, United Arab Emirates; 7 Department of Pharmacodynamics, Semmelweis University, 1089 Budapest, Nagyvárad tér 4, Hungary

**Keywords:** Selegiline, Aged-rats, Semen, Dopamine, Testis, Sperm, Testosterone, Cortisol

## Abstract

**Background:**

Selegiline is used to treat Parkinsonian patients. Other indications of its use have recently been discovered.

**Objective:**

Scouting special and beneficial side effects of selegiline treatment.

**Method:**

Two-year old male Wistar rats were daily treated with 0.25 mg/kg of selegiline s.c. (subcutaneous injection). The rats were sacrificed following a four-weeks’ treatment.

**Results:**

Mass of testes, number of sperms, progressive motility of sperms, and their viability definitely increased.

**Conclusion:**

Selegiline can successfully be used to stop/counterbalance certain symptoms of aging.

## 1 INTRODUCTION

Ecsery *et al* [1] were the first to describe the synthesis of racemic E250 (deprenyl) in 1962, while its levorotatory form (L-deprenyl or (-)-deprenyl, selegiline) was prepared shortly afterwards [2]. Pharmacology of selegiline indicated its psychic energizer effect [3].

Peculiar pharmacological characteristics of selegiline (L-deprenyl, also called: Anipryl, Carbex, Emsam, Humex, Jumex, Yumex, etc.) were discovered by Knoll and Magyar [4], was proven as the first specific and selective monoamine oxidase B inhibitor [4, 5]. Selegiline has been used in clinical practice all over the world for the treatment of patients having Parkinsonism [5, 6].

The original indication of selegiline has had its renaissan. Gordon *et al* [7] carried out their classical experiments on rats. Transdermal selegiline was successfully used in a double-blind and placebo-controlled study in hospital outpatients having major depression [8, 9].

Knoll [10] and Knoll *et al* [11] experimentally detected relationship between sexual behavior and longevity using both male and female rats.

Preliminary pharmacological experiments aimed at the use of selegiline in the possible improvement of human reproduction power. The initial process of reproduction (coitus or sexual intercourse) in some mammalian organisms contain two steps. One of them is when a male approaches a female, they copulate so that the sperms of the male enters the sexual organ of the female. Dalló [12], Dalló and Held [13, 14] made up and carried out the first experiments on how to influence sexual behavior of rats using drugs. Knoll, Dalló and others performed *in vivo* experiments on rats to show individual differences in sexual activity and also how it can be improved by treatments with selegiline [15-23]. Dalló *et al* [18] carried out their experiments using more than 200 male rats, and found aphrodisiac effects when treating the rats with 0.25 mg/kg of selegiline. The aphrodisiac effect could be observed in sexually sluggish or non-copulatory male rats [17]. Knoll concluded that improvement in sexual activity was produced in the process of a special activation of the dopaminergic system. Both the copulation process and the quality/degree of copulation mainly depend on sexual performance of male rats that can be either sexually inactive (that is “low-performing”) or highly active (that is high-performing). In the case of young adult male rats, Knoll [18, 24] deduced sexual performance from the level of the basic activity of catecholaminerg neurons, especially releasing dopamine in the *tuberculum olfactorium*, *corpus striatum* and *substantia nigra* in the state of resting [18]. Selegiline, which stimulates dopamine release on the one hand, on the other hand, mainly saves the released dopamine from degradation by the monoamine oxidase enzyme.

Knoll, Dalló and co. [10-24] classified male rats based on their copulatory behavior. Certain (1) sexually inactive male rats did not show any intention to mate sexually receptive female rats, (2) other male rats show solely intermissions (without ejaculation), (3) sexually sluggish male rats show at least one intermission without ejaculation, while sexually active male rats achieve complete sexual activity including intermissions and ejaculations. The intermission capacity of rats did not decrease significantly, however, the ejaculation ability of about 50% of these subjects decreased after 5 to 24 weeks, and practically stopped thereafter. By the treatment of male rats with s.c. injection of 0.25 mg/kg of selegiline for 20 weeks, their ejaculation ability was restored. Life span of both male and female rats was significantly increased. The average life span of the treated 66 male rats was 191 weeks, while that of the controls was 147 weeks. Similar results were obtained in the case of ovariectomized female rats treated with selegiline for 25.1 weeks versus 13.17 weeks’ treatment of the controls. Concerning intact (non-subjected to ovariectomy) female rats, they did not show any difference whether they were treated with selegiline or with physiological salt solution or not.

Another essentially important factor of reproduction is the entire quality of sperms that is the number of sperms, their progressive motility and viability (ratio of live versus dead sperms).

Clinical observations by Urry *et al* [25, 26] showed coincidence between low testosterone levels and definitely low sperm concentration. Urry *et al* [27] found that pargyline treatment had an age-related effect on the reproductive organs of male rats.

**In vitro** effects of caffeine and theophylline on human semen quality were tested by Dougherty *et al* [28] from 10^-2^ through 10^-6^ M concentrations without any change in sperm motility and viability of sperms.

Recent publications by Mihalik *et al* [29-31] treat the effect of selegiline on male sperms and the testes.

Tekes *et al* gave the first direct proof of penetration of selegiline into the testes of rats [32]. Following 15, 60 and 180 minutes of intraperitoneal administration of a relatively high dose of selegiline (15 mg/kg), its level in the testis was similar to those in serum. At the same time, the level of selegiline in the brain was higher than either in the serum, CSF (cerebrospinal fluid) or testis. The incorporation of selegiline in the brain can clearly be seen in the pictures of whole-body autoradiography taken 15 and 60 minutes following i.p. administration of radiolabeled selegiline, as well as the determination of selegiline levels using reversed-phase HPLC [32].

Similar results were found when using a rabbit model [33]. The selegiline level was similar or higher in the testes than in the brain at least 30 minutes following intravenous injection.

Zieher *et al* [34] determined a definite decline (down to about 1%) of the level of several biogenic amines through 120 days of their lives in the rats’ testes. The concentration of dopamine decreased from 22.16 µg/g to 0.174 µg/g). The determinations were done using the fluorimetric method of Carlsson and Waldeck [35],

Contrary to the effect of selegiline on the physical/copulatory activity of rats and on the brain’s dopamine concentration, the concentration of deprenyl in the testes did not show any definite increase following a four-weeks’ treatment using selegiline in aged rats.

This paper is meant to show how selegiline treatment influences the “quality” of sperms following chronic treatments with their usual therapeutic dose.

## 2 MATERIALS AND METHODS

### 2.1 Materials

**Fig. (1):**
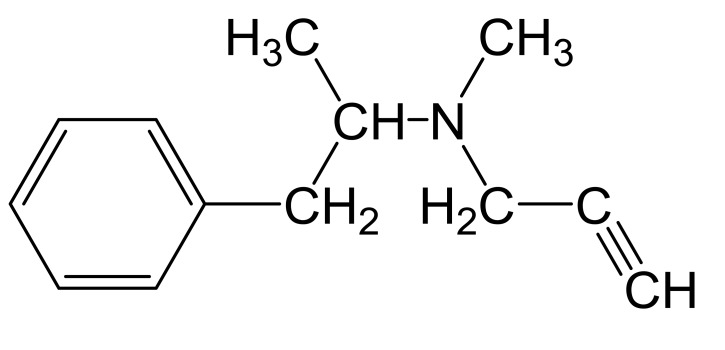
The chemical structure of selegiline.

Selegiline (Fig. **1**) was the kind gift of Chinoin Chemical and Pharmaceutical Works(Budapest); its present name is Sanofi-Aventis/Chinoin (Budapest, Hungary).

### 2.2 Animals

Male Hanover Wistar rats of two years of age were supplied by Toxicoop (Budapest, Hungary).

### 2.3 Methods

#### 2.3.1 Treatments of Rats

Ten rats (five treated and five controls) were o.c. treated with a 0.25 mg/kg daily dose of selegiline for four weeks. Treatments were performed according to the experimental protocol approved by ethical committee of ANTSZ (Budapest, Hungary), permission number: 1810/03/2004. The experimental conditions conformed to 86/509 EEC (European Economic Community) regulation. The control group received only the physiological salt solution.

#### 2.3.2 Preparation of Tissue for the Analysis of Sperm Motility and Morphology

The testes and epididymes were removed and the cauda epididymes were ligated at autopsy and kept in PBS (Phosphate-Buffered Saline) until semen evaluation. Following the separation of the complete epididymis, the testicles were weighed. The epididymes were placed in Petri dishes containing 0.5 mL PBS with 10 mg/mL of BSA (Bovine Serum Albumin). Cauda epididymes were minced with iridectomy scissors, by five deep cuts. The Petri dishes were covered and incubated at 37 °C for 10 minutes. Prior to the assessment of sperm motility, 10 µL of supernatant was dropped at the prewarmed slides and covered with a lid. The sample was held at 37 °C while the velocity parameters, including the progressive motility of minimum 100 sperms at 10 fields, were observed. Progressive sperm motility was evaluated using the standard method of Bearden and Fuquay [36]. The mean of the ten estimations was used as the final motility score.

The total sperm number was determined by using a hemocytometer. Approximately, 10 µL of the diluted semen was added to 390 µL of Hank’s Balanced Formaldehyde Solution. Ten microliters of semen, fixed in formaldehyde were transferred to the counting chamber of the hemocytometer and the cells were counted with the help of a light microscope (200x). The sperm cell number of ten large squares multiplied by the dilution rate gave the original sperm cell count.

Sperm cell morphology was evaluated by stain of Eosin-Nigrosine and Spermac Stain at a 400x magnification (FertiPro N.V., Beernem, Belgium) as it is prescribed [36].

#### 2.3.3 Tissue Homogenization and Hormone Concentration

Small pieces of each testicle and adrenal gland were precisely weighed by analytical scales. The pieces were homogenized with PBS by a tissue homogenization (Janke&Kunkel Ika-Werk Ultra Turrax). The homogenates were centrifuged at 4000 rpm for 20 minutes. The supernatant was stored at - 20 °C. The stock-dilution of tissue was 1g/10ml at the homogenization which was 1:5 diluted individually for the determination of tissue hormone concentrations. Serum and tissue cortisol and testosterone concentrations were measured by ELISA (enzyme-linked immunosorbent assay, DRG International, Marburg, Germany). The intra-assay variability (CV) of cortisol ELISA was 3.3%; the inter-assay CV was 6.1%. The intra-assay CV of testosterone ELISA was 3.21%; the inter-assay CV was 4.74%.

The testes were halved, homogenized in a solution of 0.1 M trifluoroacetic acid, centrifuged and their dopamine contents were measured by HPLC according to Rao *et al* [37]. The analysis of the samples was performed on a JASCO HPLC system consisting of a 4180 analytical pump, a 4050 autosampler unit and an Antec Decade Elite electrochemical detector (ABL&E-JASCO Magyarország Kft, Budapest, Hungary). The sample components were separated using an isocratic method. The mobile phase was slightly modified from that of Rao *et al* [37] containing 6.8 g of sodium acetate, 5.9 g of citric acid, 48 mg of sodium ethylenediaminetetraacetate, 850 mg of octanesulfonic acid in 900 ml of water, at pH=3.6 and 100 ml of methanol. The stationary phase was a Recipe ClinRep analytical column for catecholamines in the plasma, catalogue Nr. 2030 (Unicam Magyarország Kft, Budapest, Hungary) mounted with a Phenomenex SecurityGuard™ C8 cartridge (Gen-Lab Kft, Budapest, Hungary). The flow rate was 0.9 mL/min and the stationary phase was kept at 25 °C. 20 µL samples of aliquots were injected, kept at 5.0 °C during the analysis, and the run time was 15 min. System control, data acquisition and evaluation were conducted employing the ChromNav 2.0 software (ABL&E-JASCO Magyarország Kft, Budapest, Hungary) and Microsoft Excel 2010.

## 3 RESULTS

Important characteristics of sperms show definite improvement in their progressive mobility (Table **1**).

The average mass of the testes, number of sperms and viability (live/dead sperms) were also increased relative to these characteristics of rats in the control group. Cortisol content in the serum increased, while that in the adrenal gland significantly decreased. Selegiline treatment definitely affected the testosterone level in the sera, the adrenal glands and testes, whose levels decreased (Table **2**).

## 4 DISCUSSION

Hársing and Vizi [38] did not show significantly increased dopamine release following acute treatment of rat striatum using a therapeutic dose of selegiline. A four weeks’ (chronic) treatment [39] significantly increased both the dopamine content and KCl facilitated dopamine release from rat striatal slices. Certain recent experiments have shown that not only selegiline, but also p-fluorodeprenyl and rasagiline significantly improve the “quality” of sperms. Para-fluorodeprenyl presents the most pronounced improving effect, while the effect of rasagiline proves to be the mildest.

Zieher *et al* [34] raised the question why the concentrations of the determined biogenic amines (dopamine, histamine, norepinephrine and serotonin) declined in the testes. However, no definite answer was given except that Zieher *et al* [34] suggested that further experiment should be done.

## CONCLUSION

Our results do not correspond to those of Urry *et al* [25, 26], and Wanichacheewa *et al* [40] as low testosterone levels and sperm concentrations were found inversely proportional. To describe and characterize sperm motility Yanagimachi [41] and Bavister *et al* [42] used catecholamines (isoproterenol and norepinephirine) **in vitro**. Ramirez *et al* [43] published the presence and function of dopamine D2 receptor in boar sperms, which is involved in the viability and motility of sperms. The presence of 100 nM dopamine definitely increased sperm viability 1 hour through 4 hours, as calculated by them from 10 parallel experiments using student t test.

## Figures and Tables

**Table 1:** Mass of testes, progressive motility of sperms, number of sperms and viability of sperms of control rats and that of sperms of selegiline treated rats.

Rat (No.)	Mass of Testes (g)	Progressive Motility of Sperms (%)	Number of Sperms (x10^6^) in mL	Viability (live/dead)
1 (control)	172	58.9	132	82
2 (control)	185	61.3	147	91
3 (control)	181	52.2	069	73
4 (control)	216	71.3	178	81
Mean	188.5±13.75	60.925±5.375	131.5±31.250	81.75±4.75
5 (treated)	179	78.6	143	92
6 (treated)	282	81.8	109	89
7 (treated)	199	79.5	209	96
8 (treated)	195	77.2	232	94
Mean	213.75±	79.275±	173.25±	92.75±
p =	0.180257	0.044423	0.728	0.172

**Table 2:** Cortisol and testosterone in serum, homogenate of adrenal glands and testes of control and selegiline treated rats.

Rat (No.)	Cortisol			Testosterone		
	Serum (nmol/L)	In Homogenate		Serum (nmol/L)	In Homogenate	
		Adrenal Glands (ng/g)	Testes (ng/g)		Adrenal Glands (ng/g)	Testes (ng/g)
1 (control)	27.078	247.992	59.079	4.48	1.155	74.469
2 (control)	121.629	501.210	42.755	8.786	0.788	54.162
3 (control)	283.618	427.236	41.197	2.997	2.001	7.199
4 (control)	46.72	232.677	44.420	14.348	1.114	49.409
Mean	119.76± 82.86	352.28± 111.94	46.86± 6.10	7.65± 3.91	1.26± 0.3682	46.31± 1955
5 (treated)	166.944	248.552	43.940	1.879	0.870	16.927
6 (treated)	54.843	106.283	56.808	6.918	1.074	23.231
7 (treated)	142.744	476.313	47.804	4.255	1.206	23.768
8 (treated)	197.448	134.774	26.454	3.522	1.001	16.670
Mean	140.49± 42.82	241.48± 120.95	43.75± 8.64	4.14± 1.44	1.037± 0.1022	20.149± 3.3505
p =	0.3194	0.7081	0.4947	0.1794	0.0595	**0.0084**

HPLC analysis of the testes did not show any dopamine content over 5 pg/mg of tissue.
